# Supplementary material for: Two neuronal peptides encoded from a single transcript regulate mitochondrial complex III in Drosophila
Source: eLife. 2022 Nov 8;11:e82709. doi: 10.7554/eLife.82709 (PMC9681215; doi:10.7554/eLife.82709)
Supplement: Supplementary file 1. [file elife-82709-supp1.docx]

Supplemental File 1

Genomic sequences of *sloth1* and *sloth2* homologs

**BOLD = Coding sequence**

Red = *sloth1* homolog

Blue = *sloth2* homolog

>Dmel_sloth1-sloth2(CG32736-CG42308)

AATCGAACAGCTGATTGCTGCGAACCGGAACAAATGGAAATTGTATCGTGAGgcaagtggagtttcccctttacttttggcaaataataaataaacaaaggaacaagcctaaacattttcaattaaaccatatacagAACTAACGCACACATGTGACGGAGGCAATACACAAACACGGCACCTTTGAATCTCGCCTTAAAATTGGCGAAACCAACACGGAATTATATAACCGCCGGCTGAAAACAC**ATGAGTCCGTACAGCGGATCCGTGCGTCGTCTGCTGGACAGTTGGCCAGGAAAGAAGCGCTTCGGTGTCTACCGCTTCCTGCCGCTCTTCTTTTTACTGGGCGCCGGCCTGGAATTCTCCATGATCAATTGGACAGTGGGCGAGACCAATTTCT**gtgagactgctacgcttaaaaccttacttttatttactaatacggaatcttttccatgcag**ACCGCACTTTTAAGCGCCGCCAGGCGAAGAACTACGTGGAAGAGCAGCAGCATCTGCAGGCGCGAGCCGCGAATAACACCAACTAA**GCAAA**ATGCCCGCCGGAGTTTCCTGGGGCCAGTACCTGAAATTCCTCGGCTGTGCCCTGGCATCCATGATGGCCGGATCGCAGGCTGTTCACCTTTACTATAAGCCTCTGGAGGACTTGCGCGTCTACATCGAACAGGAGCAACACAGCACACAGGTGGATCCCACCGCAAAGCCACCGGAATCTGCATAA**CACTGTGTACTAGACAAGTTATTGGTGACTAAAGCTATTTAAG

>Choanoflagellate_Salpingoeca_urceolata_sloth1-sloth2_comp15074_c0_seq2

TTCACTTTCGTTTTCTTACTGTTTCAACGTTGCGACTGTGCTCTTCGGCTTCACGTGTTCTTGCACCATCTGCTGTGGCACCCATTCAGCGCAGAGTTCAGCGGTCCACGCAGTGGCAGCGGGCCAGGACACCACTTCTGCTTGGGTACCTCTA**ATGCCGCGTTCGTTTCCGCAAATTGCGGCGCGTGTGGTGCCTGTGTCGTTTGCTCTTGGCGCGTTTATGGAATGGTTCATGCTCAACGTTCAAATTGGCCACGAAACCTTTTATGACACTGCAGTGAGGCTGGAAGCAAAGCGACGGTTTGAACAACAGCAAGAGGAGCAGCAAAAAGCTAGCAACGACCCTTCGTCCGACTCACCGCCGCCAGCAGCATCCTAA**GAGTTGTTTGCTTCCTGAAGTAGTTTTAGTTTGTACCTGTTGTTTTTCGTTAGTTTTTTTGAAGGTTCCTTCACGTCCAGCACC**ATGCCGTTTGGTGTTTCCATGTCTCGGTACGTGGGTGTGGTCGCACTTACCCTCGGGTCCATGCTGGCCGGTGCTTCCACCGTACACTACTTCTACCAGCCCGACCTGACTGTGCCCACCGAGCCTCCTCCGGCGCCGGATTCCGTGTTGAAAAAGCCACGGATAGCCTTGGTGTCGCCACGGCAGCGTGCGACGGGAGAAGCAGACGATGGAAAACAGTGA**CCGGTCTTATGCGTGATTGGTATTAAACACATGGTCGTGTTCAAGATGAGGTTGTTGGTTGCCAGTGCCGCGGAAAACCCGCAACATGGGCGCTTGTCCCAATACGTTTTTGCTGTGGGGTGTTCGTTTTTCTTTTTCCGGTTGGTTGTTTCATCCTCATTCGCCACGCAGCAGCAAAAAGCAACAAGTCAACTCGATTG

>Lamprey-Petromyzon_marinus_sloth1-sloth2

TTTCTGTCTGTGCCCGCGTGTCTCTGTGTCCACATGTCTGTCTGTCCATGTGTCAGGGGGTGCAGCGGGCGAATGGGCG**ATGGTGTTCTTCAGCAGCGCTCTCGGGAGGATTCTCAGTAAAGTTCCCGGAGAGAAGAGGCTGGGTGTCTATCGGTTCCTGCCCGTGTTCTTCGTGATTGGCGGTGCCATGGAGTGGATCATGATTAACATGAGAGTCGGCAGAGAGACCTTCT**GTGGGTACCACGCAGGGCTTCATTATTTCTCACTGAAATATTTTCCGGGTGACCGGTAGACTGGAGTTGGTTGCACATGATTAGTATCCACGGCCTGGTAGCCCTGAACAGCGCCTACACTGGAATCGGGACTCGCATGCCACGCGTTTGACTCTTCGTTTGACCCTTCGTTTGACCCCGGCGTCCCATTATTTACCTCTGACACCGCATGCTCACCATCGAGTGCGACTAACCGCACGCGACGGCGCGCTGTTTCTTTCAG**ACGACGTCTACAGACGCAAGCAGTCGGAGCGCCGTTACCAGCAGCGCCTCGCCGAGACCTCGCAGTCCAGCGGTTCCAACTAA**GAGTCTCGCCTTTCTCGAACAGACGATCGACTCGGTCACCACCCCACACGTCACTCCGTCTCCTCCCCCCCTTCCCGCCGTTGTTGCTGCCGCCGCTGCCACCACAACCGACTTGCGCTGCTTGCGTAGAAGCTACGGGCGCAAAGAACTGACGGCTCGCACTGGGCCGTGCGTGAGACTTTCGGAGCGAGGTTGTTGACA**ATGCCGGCGGGCGTGACGTGGCCGCGCTATCTCAAGATGCTGACCGCGAGTCTCCTGTCAATGCTGGCAGGAGCGGAGGTGGTTCACCGCTACTACCGGCCAGACCTG**GTACGTGGACTTTTTTTCTTTCGTTCTCAGGAGTCCGGCTCGGGGATATAAAATGTTCACGTTATAAGCCATTTCATTGAGCTATCATATGTGATAACCAGGTCGCTTCTGAAAAAGAGCTAAATTACTCATTGGGCCTTACCTAGTAAAAAAAAATCCCACTGAGTGTTTTCCGGGTCTCTGGTTAAACCCAAGAAGGTGACTCGCAGTAGCCGCAACCATAGCGAAGGAGGTATACTTGATGTGGTGTGTTGGGTGCAGAAATACAGGACCCCAAGAGACGCTGCTACCCGTAGTGTATCTGTGTGGATATCCGGTGTTAATTGCCATGTAAGAGTGGGTAAGAGGATATTTCGATAGTACCACCCCAACAGGGATAAAGAGGGGTTTCCACCGCATTGCTGTTGTTCACTGTTGCGGTTTCCCTCCCACACAG**AGCATCCCTGAGGTTCCGCCAGCGCCGGGGCAACTGCAGACGCGGCTGTTGGGCATCGAGGGCACAACGGGGACACCACTCAGTGGCACCAGGGCTGCGGAGGAGG**AACGCAGCCATCCCTCGTGACGGCGTCCACTCCCTCAACCTCGAGCACGTGCACGTGCACGAGTTAACGCACACACGAACATGCACAGGAGGCACAGCACATGCACAGAATGTTATACCTCCTTCACGATGGTGAATCAAAAACGATAAGACTTTTTATTTTAC

>seasquirt_ XM_018812254.2_sloth1-sloth2

TTCAAAACAGAACAGTTATCAAATGTATTATGTAAAAATGCAGTTGAGTATATGAGTAAGCCAGTAGTACATAATATAAACCATACCCTCGGTCTGGAGCCACAAATACTTAAAACAAATACGGCTAATACTTTTTGTAATATTCTAGTAACAAAACCTGATTTTTAAACATATTTGGCCCATTTTAGAGTTGTAAAGTATGAATTGTTTCTAGT**ATGACGTTTATTGGTCGACTGGTCCAGACATTTCTTTACTACTACCCAATAAAAAGACAAAGCCCATACAAATTCGTTCCACTGTTTTTTGCCATTGGAGCGTCTGTGGAGTGGGTTATGATAAAAGTTCCGGCTGCAGGACGAGGTGAAACATTTTACGACGTTTGGAGAAGAAATAGATCAGAAAAAGAATACAAGCAGAGAATAATTGAAGAGAAATTTCAAGAAGCAATTAAAGCAAAAGAAAACTGTGAAAATTAA**TAAGCATATATTTGGCTTGTCTTAAACTGCATTAAACACTTAATTTAAATAAATTACCTTTGAAAAAATCAATAATTTACTTTTATTATAAGTTTAAACAGTTTTTTTAGCTTGAACTTGCGTAAAGAAATTTAGGCCTAAAATTAAAAATCACCCAAAAACACTTTCTGTTCATTTAATAAGCAAAACCTTTTGTTTGATTTATTTTCCAACTGTATAATTTTGCATACCCACCACATC**ATGCCTTATGGTGTTTCTTGGCCATTCTACCTGAAAACAGTATCTTCTTCACTCATAGCAATGTTCCTGGGCTCACACAGTGTTCATATGTGGTACAGACCTGATCTATCCATACCTGAGATCCCACCTAAAAAAGGGGAGCTTCACACAAAACTTTATACAACAAAATCAGAAAATTAA**ACGAATTCATTACTTTTGTTAATGTTTTTTTGGTAACCTTAATCCAGTGTGCAGTTGTACTATACGCTTATTTTTTTTTTGGTAGCTTTGTTTCAGCTAGTTACTTGTTTTCTATCAGGTATACTGGTAATGTTTTGGTTTACATTTATTTATGAAGAAGATAAGTTTCCTTCTGCTAAGTAAAAGTTGGCATTTTAAATGTAATTCACTTTAAAAACCCATATTTCAGTTTCATTTCATAACGCTTTTTGTGTTTGATCAATTTTTGGCTGTGAACAAATTTTGTGTTTGTTTGACTCAACCTAAAAACATCTCCTTACTTATTAGGTTGACTGTATAGGGCAAAGTAGTTTTCAAACATTGTATAACTTTTCAAGATGGCCGACAACCTTAGTGAAGAATGGTGGCAAACGGCAGTTTCTGATGAAGAAGAAGGCGCAAGTGATGATGGTGAACGAAAAGAAATGAAACGTAAACTGAACGAACCGACTTCAGGAATAGTAGTTTCAGAAAACGAGGAACCAGAAGTGAAAAAGAAAAAAAGGCGGAACAGAAAAAGAATTACTGAAGCTAAGCTTCCCGATCAAGGGGATTCACCCACGATGTTACGAGATTATCTCAAACTTCACTTCAGTAAATTATCCAAGCTCGAATTTGAGGATATTTCGCTAACAGAATCCAATTTCACAGCATGCAATATCGACAAAGAACATACTACCACGTCGTATTTTAAACAAATCGCCCCCAAGTGGCATCGTTTAAGCACAGCTCACAGTCACAAGATGTCGCCTCTGATCATCGTGGTTTGTGGCAACGCACTTCGAGCGTCGAAATTTAACACAGAAGCAAAGACTTTTAAGGGCAAAGATGCAAGGTCGATAAAGCTATTTGCGCGCCACATGAAGATCGACGATCAAATCAAACTTCTGCGGGAAAACGTCATTCATTTCGCCGTCGGCACACCGGAAAGAATCCGATCTCTTATCCTACAAGATGCTCTCAGTTTAGAACACACTCGAGCGTTTGTCATCGATTGGAATTGGAGAGATGTAAAACTAAAGCGTTTAATTGACATACGAGAGGCTCGTGCGTCGTTGATGAATTTGTTAAAAGATTGCGTGATCCCAGCTTGTAAGAAACACCATGTAAAAATCGGGTTGTTTTGATTTGAATTTGTGCAAAAAATGAGGTTTTCTGACGTCATACAGGTTCAAAATTTGCTTGTGTGCATGGCCCGTTTTTTTCAGTAAATGGTTTACGTTCATGCAATAAATTGCCATTTTAAGTTAGTGTA
