# Supplementary material for: Two neuronal peptides encoded from a single transcript regulate mitochondrial complex III in Drosophila
Source: eLife. 2022 Nov 8;11:e82709. doi: 10.7554/eLife.82709 (PMC9681215; doi:10.7554/eLife.82709)
Supplement: Supplementary file 4. [file elife-82709-supp4.zip › Supplemental_File_4_-_Raw_gel_and_western_images.zip!/uncropped gel and western blots.pdf]

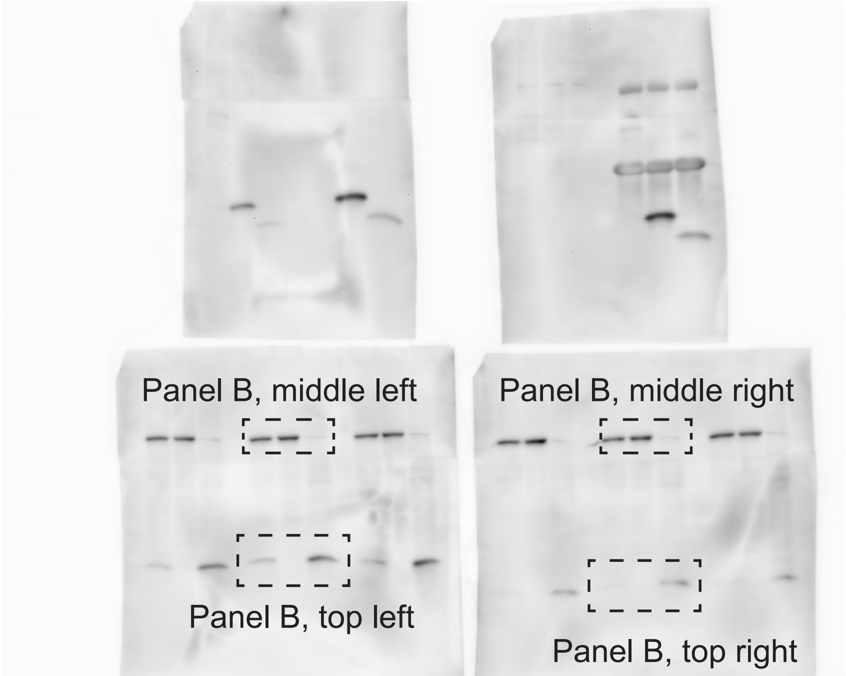

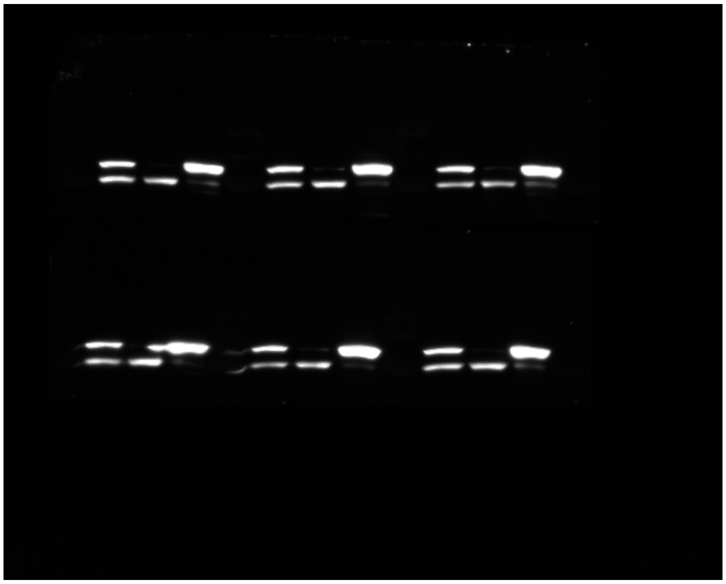

Raw image

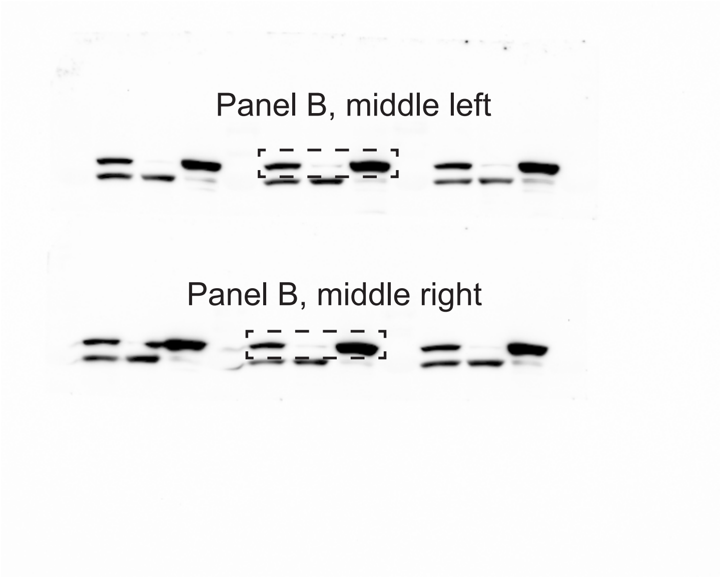

Inverted

Figure 6-source data 1-2

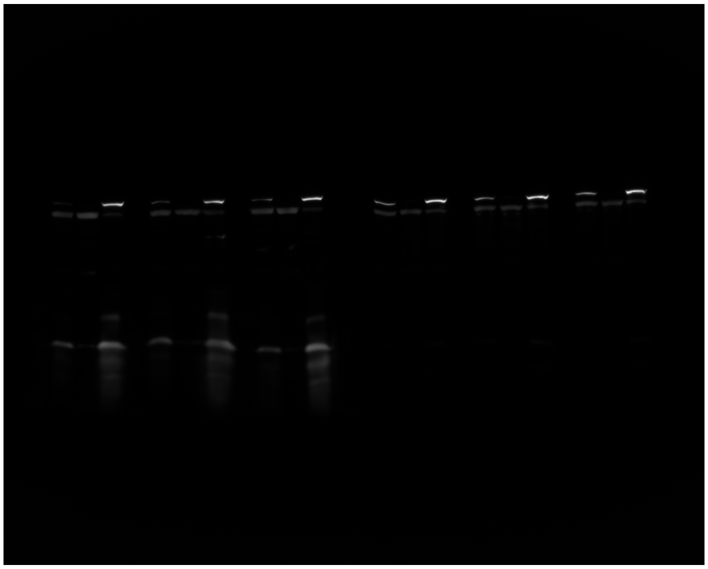

Raw image

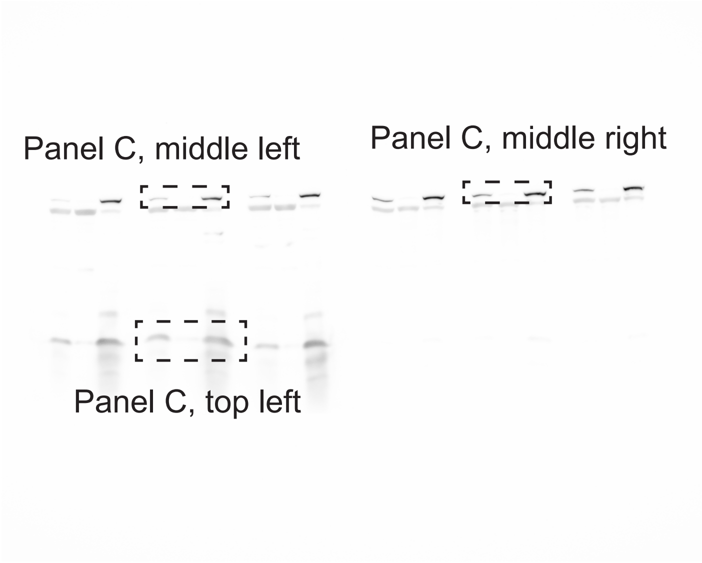

Inverted

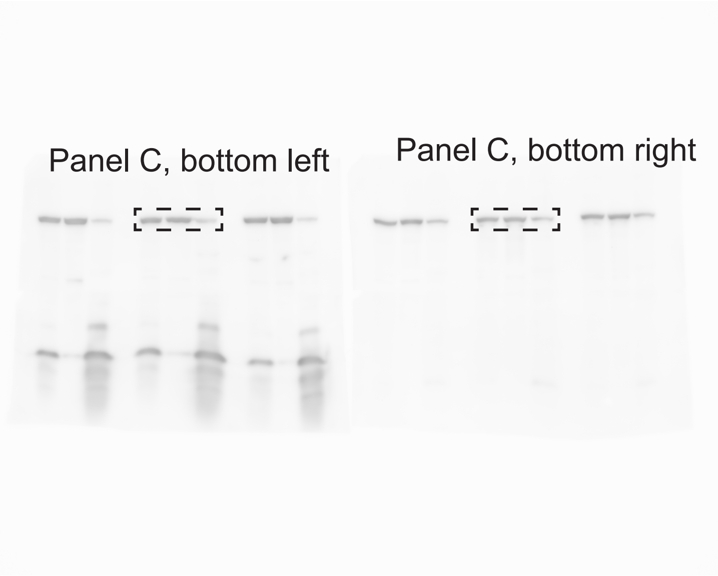

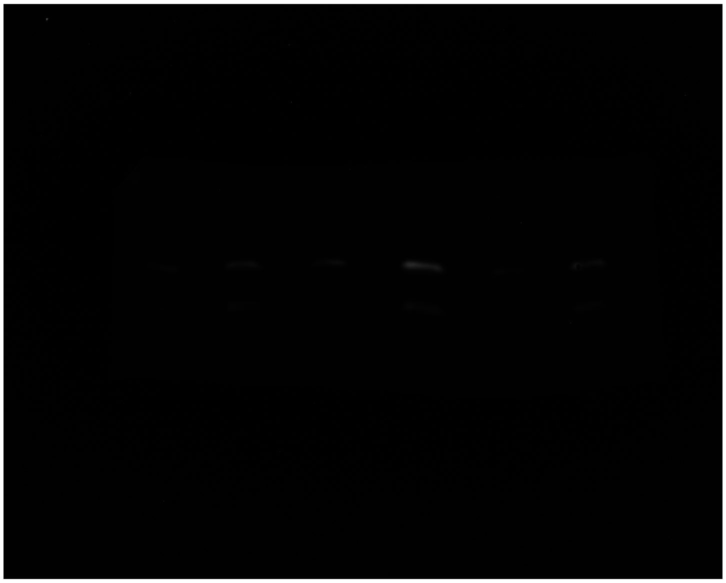

Raw image

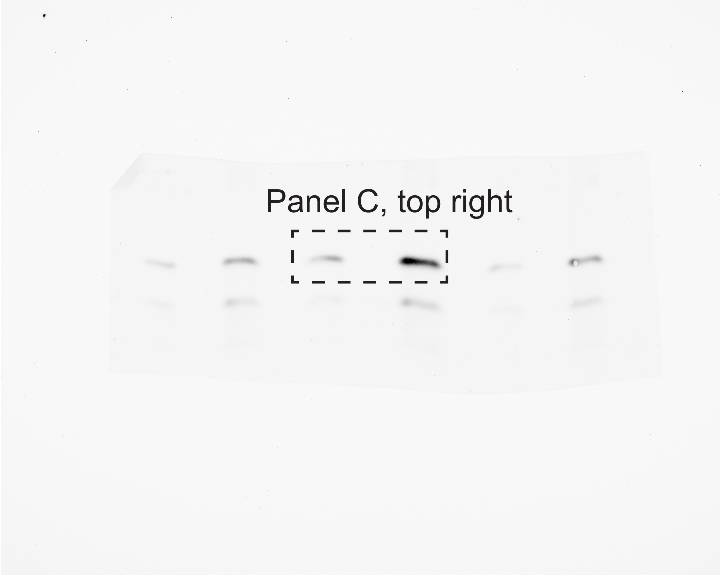

Inverted

Figure 6-source data 3-5

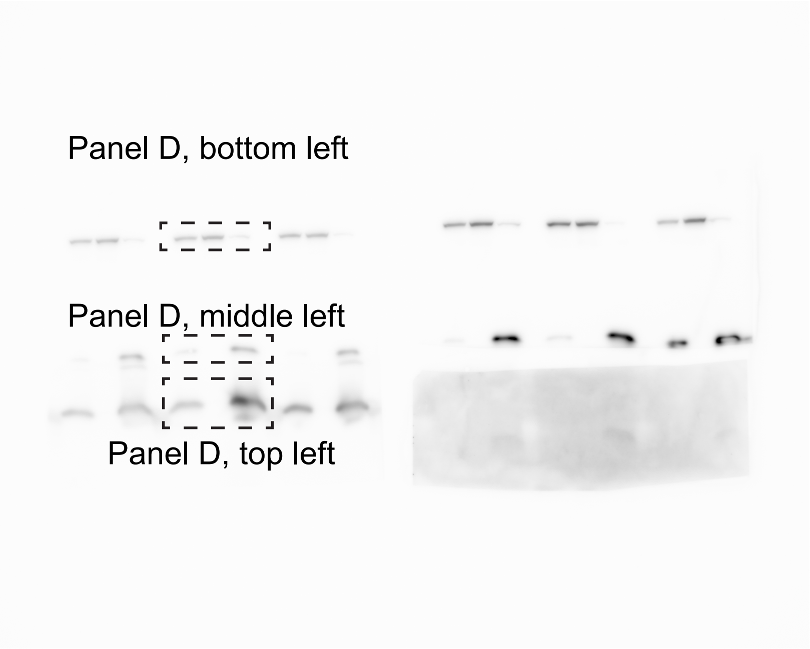

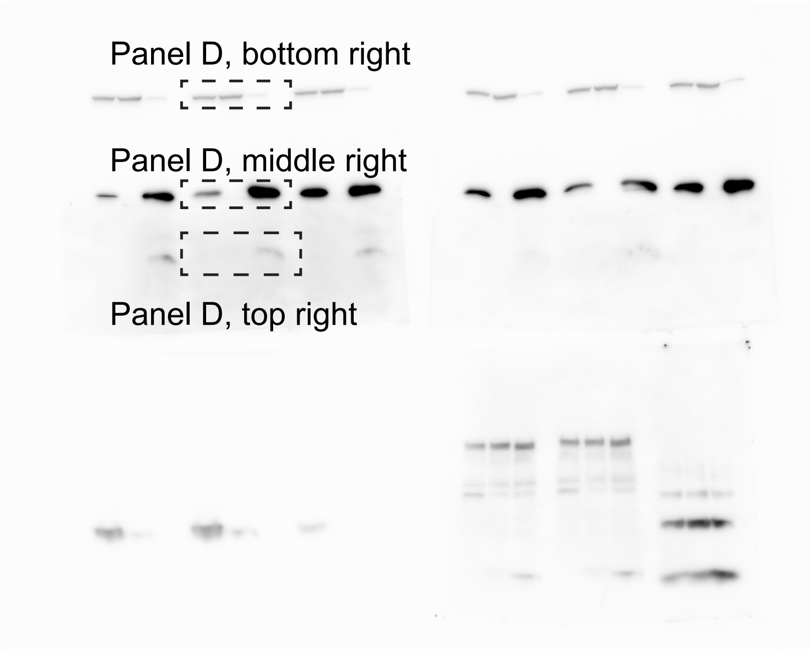

Figure 6-source data6-7

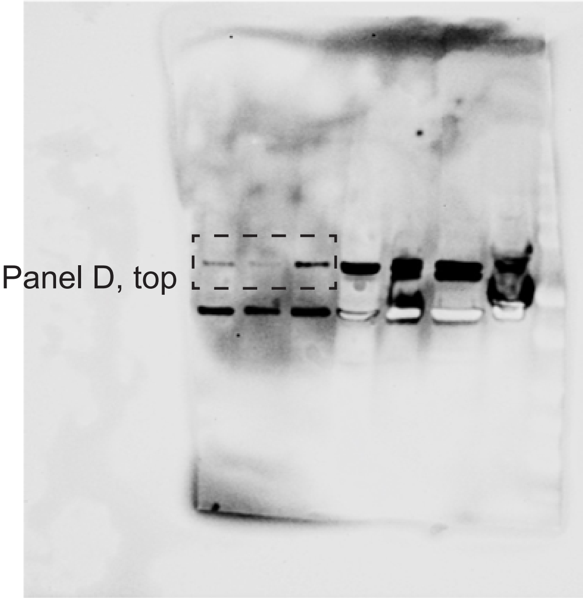

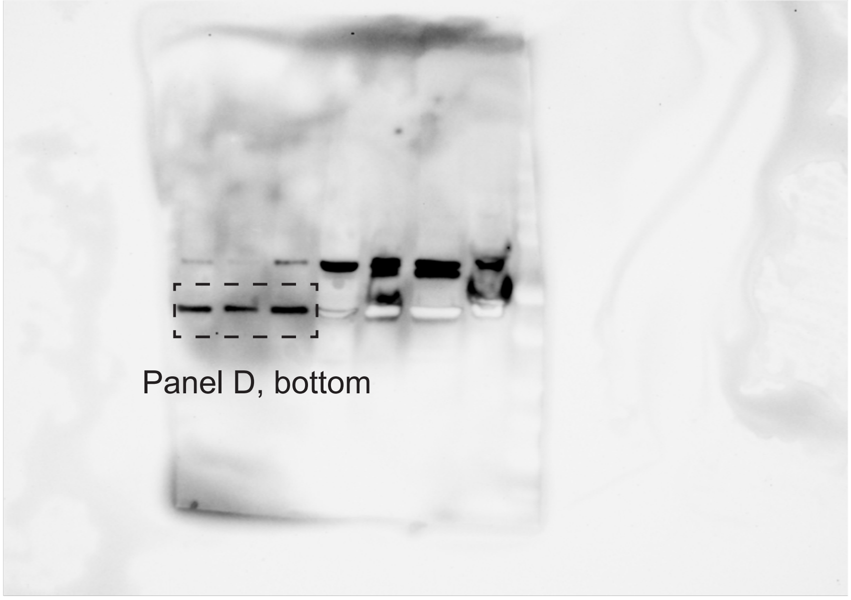

Figure 7-source data

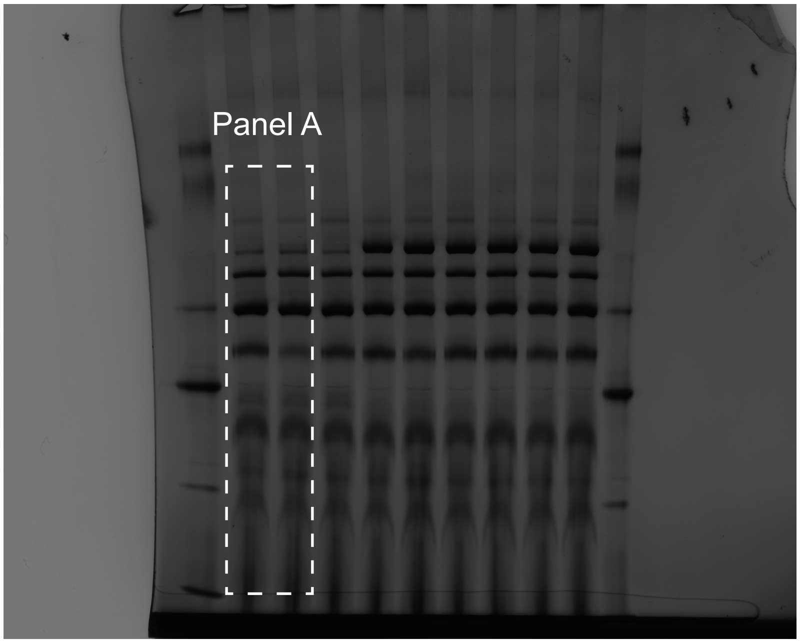

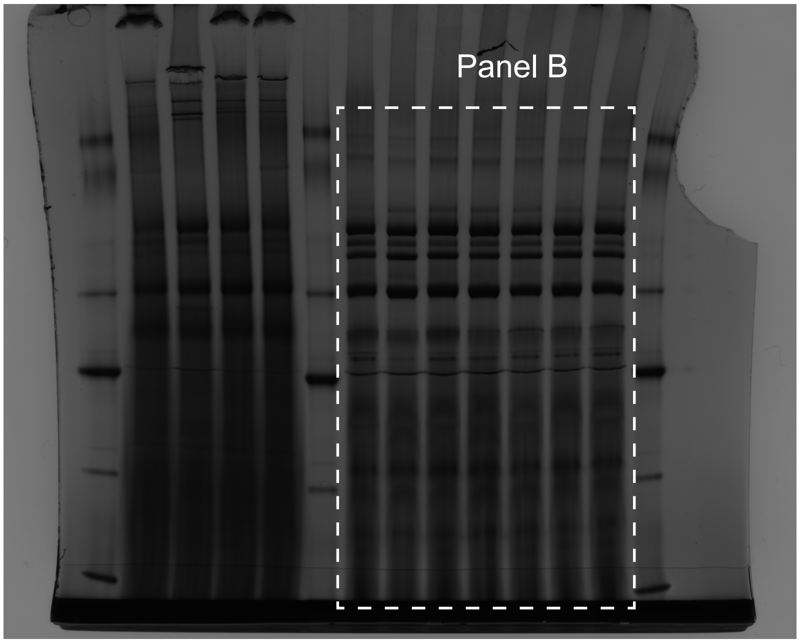

Figure 8-source data

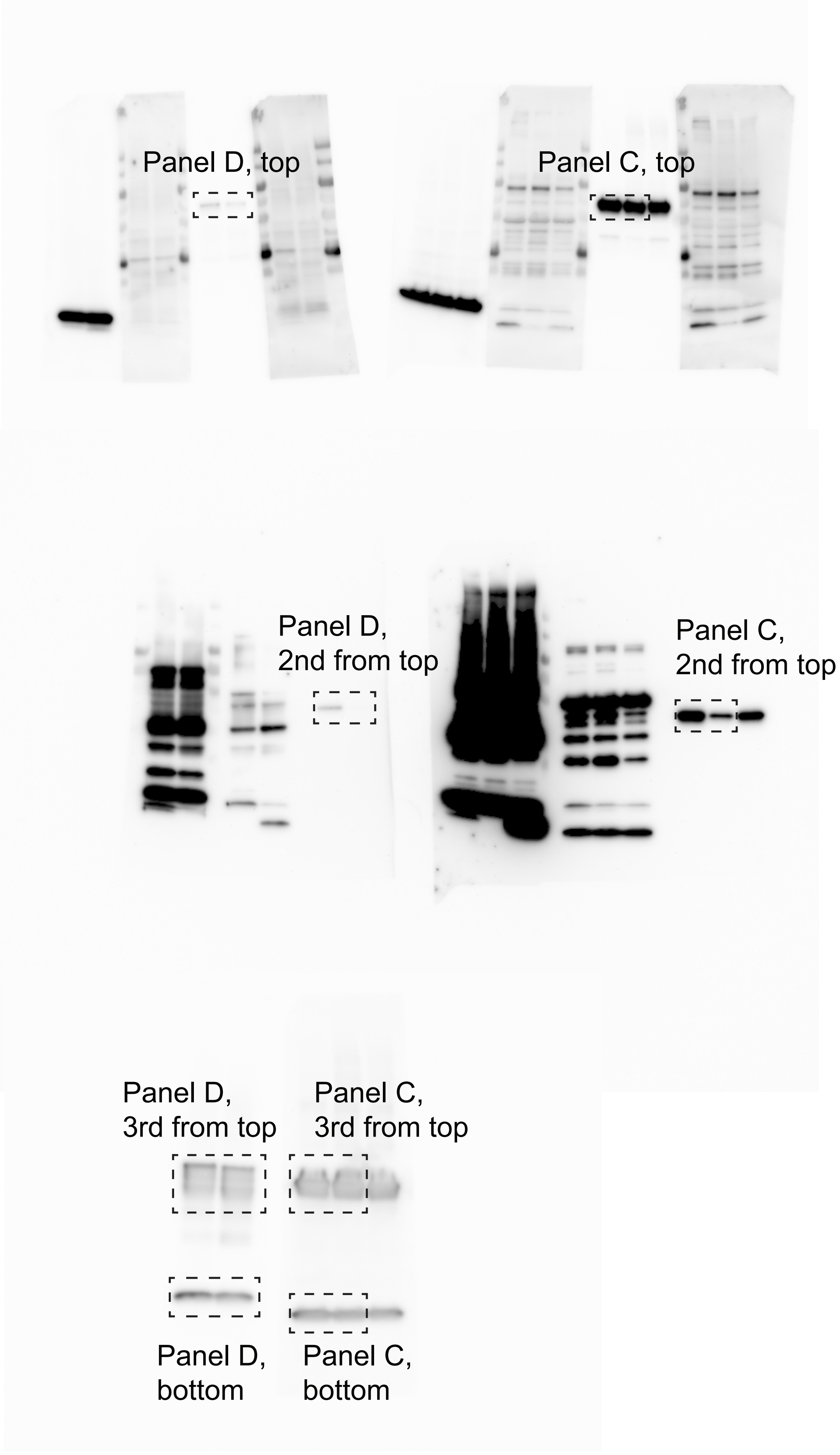

Figure 8-source data 3-5

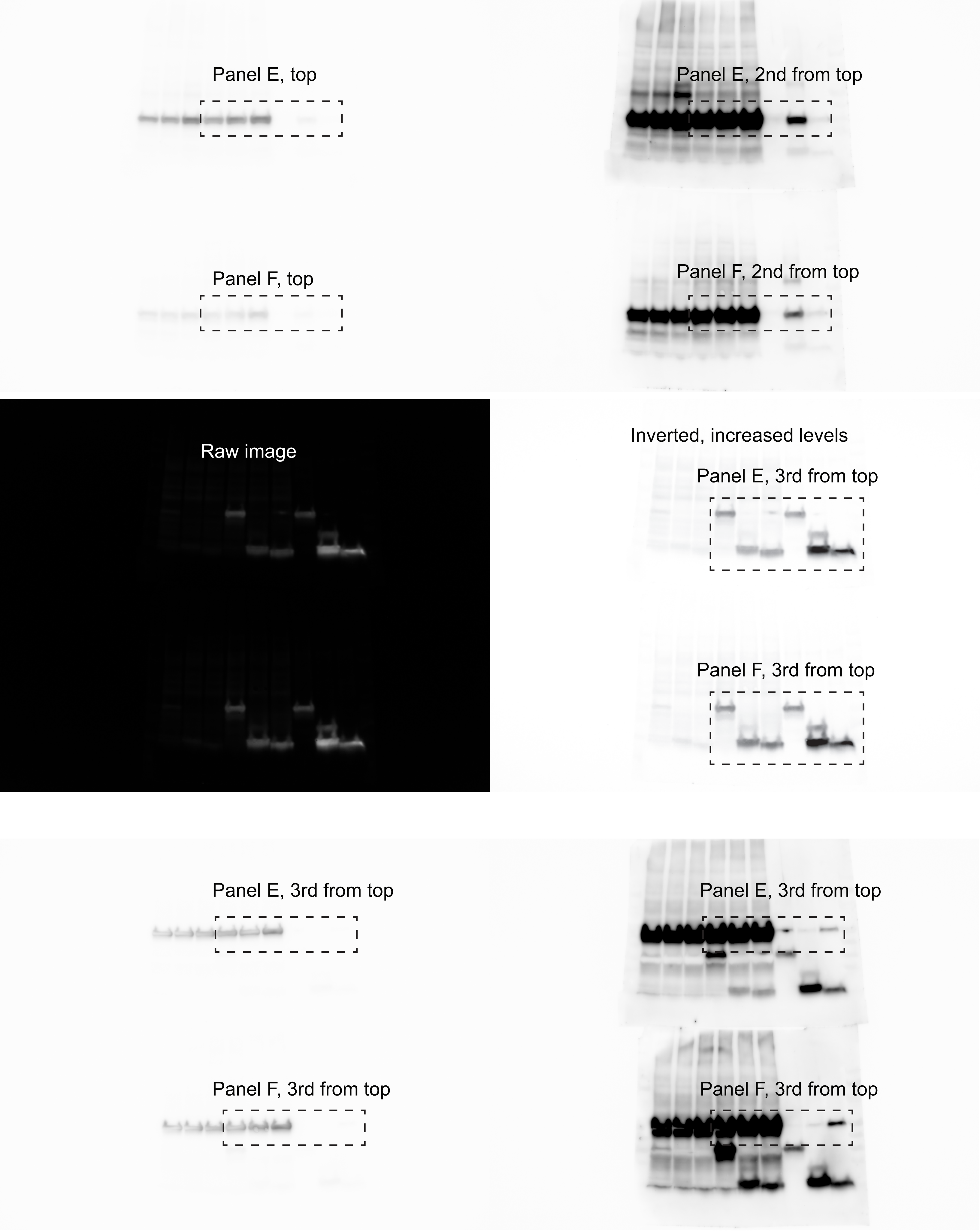

Figure 8-source data6-10

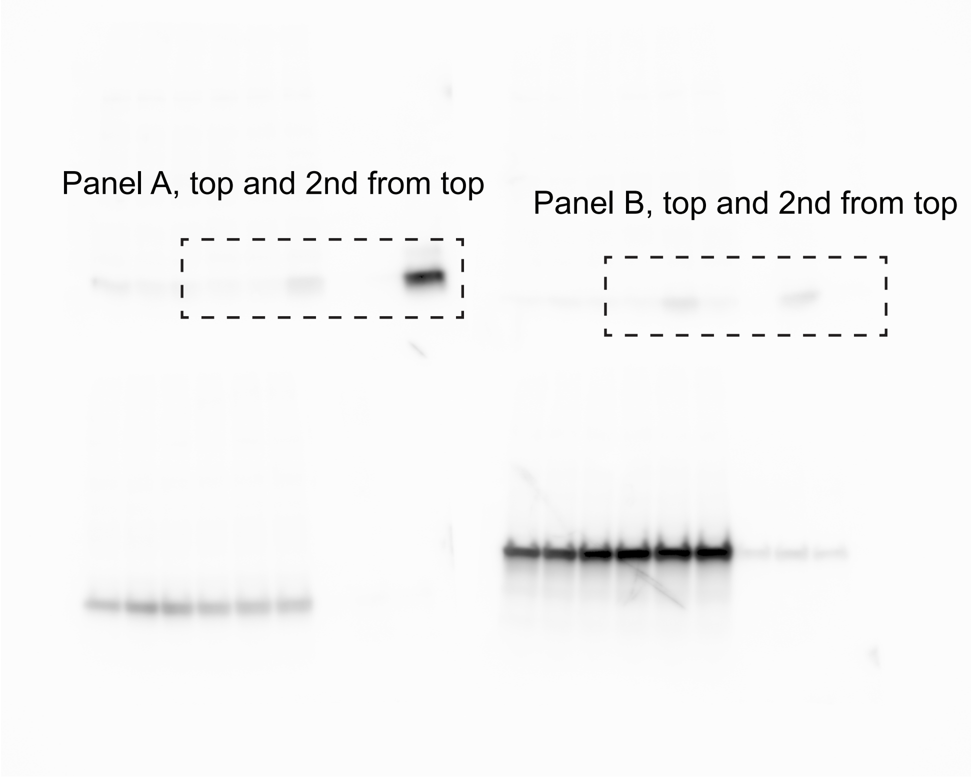

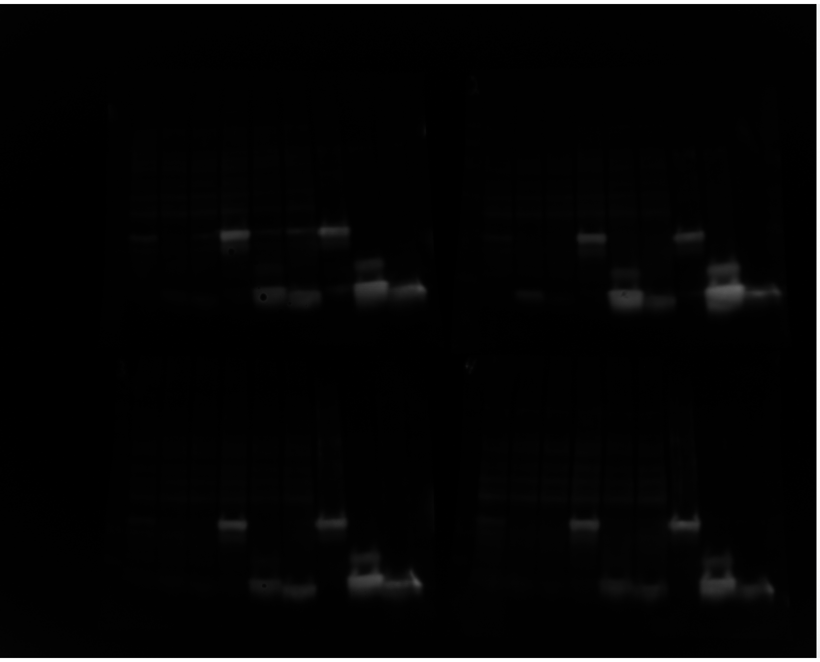

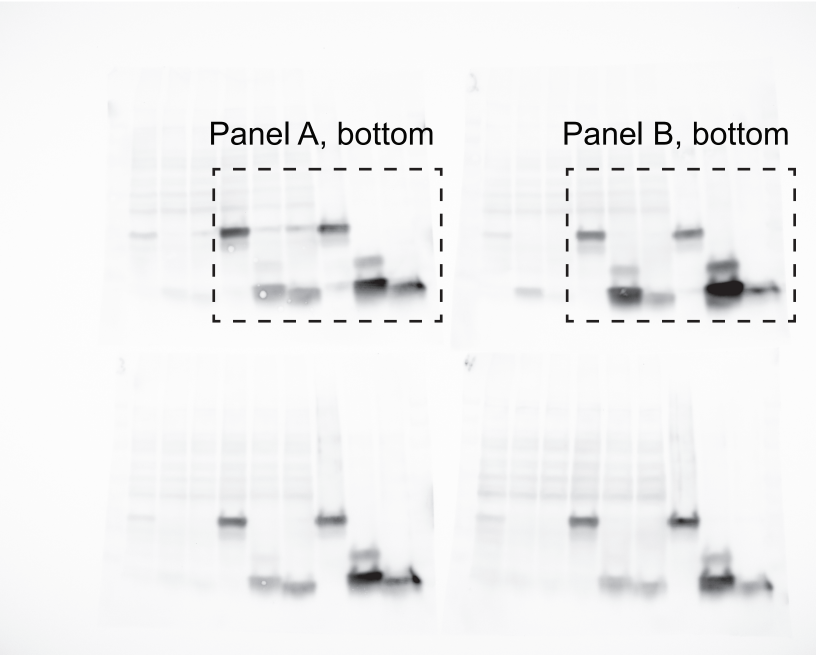

Raw image

Inverted, increased levels

Figure 9-source images1-2

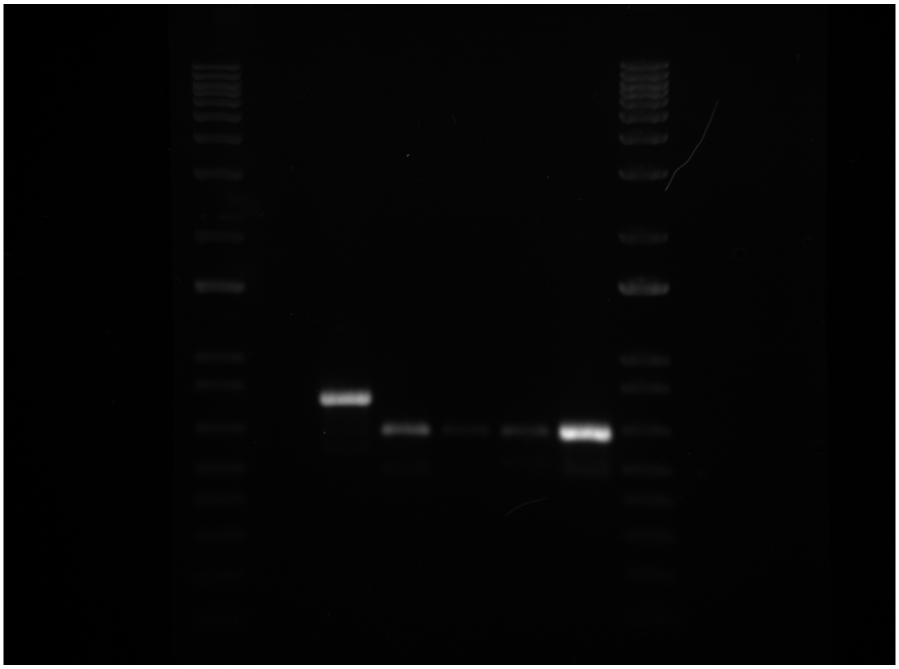

Raw image

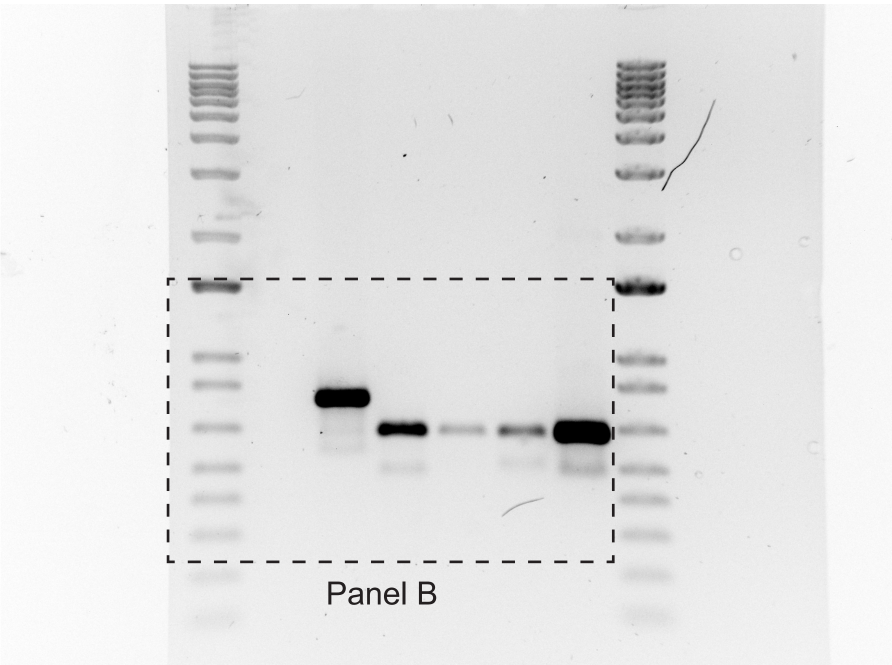

Inverted and increased levels

Figure 1-figure supplement 1-source data

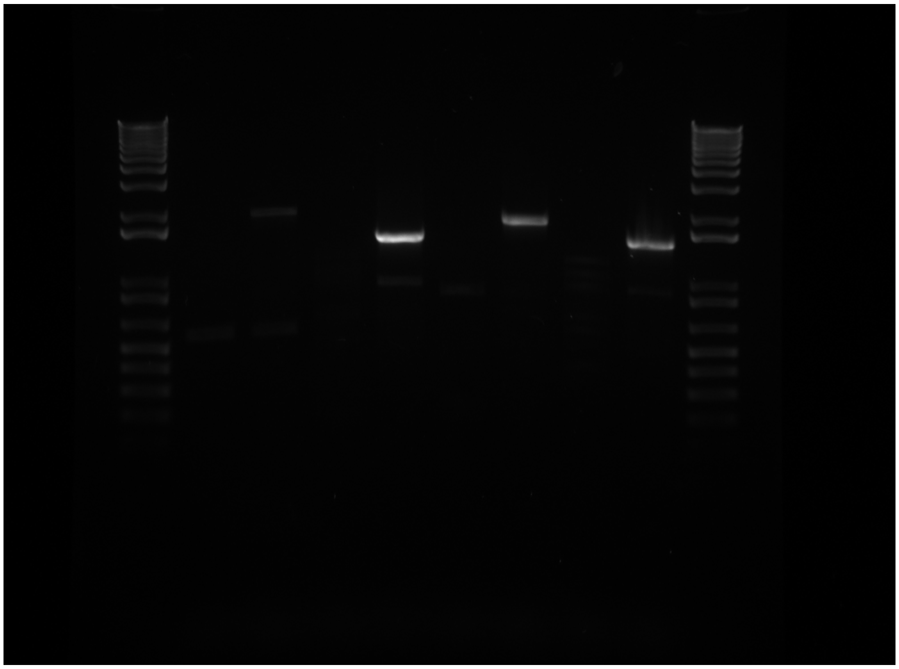

Raw image

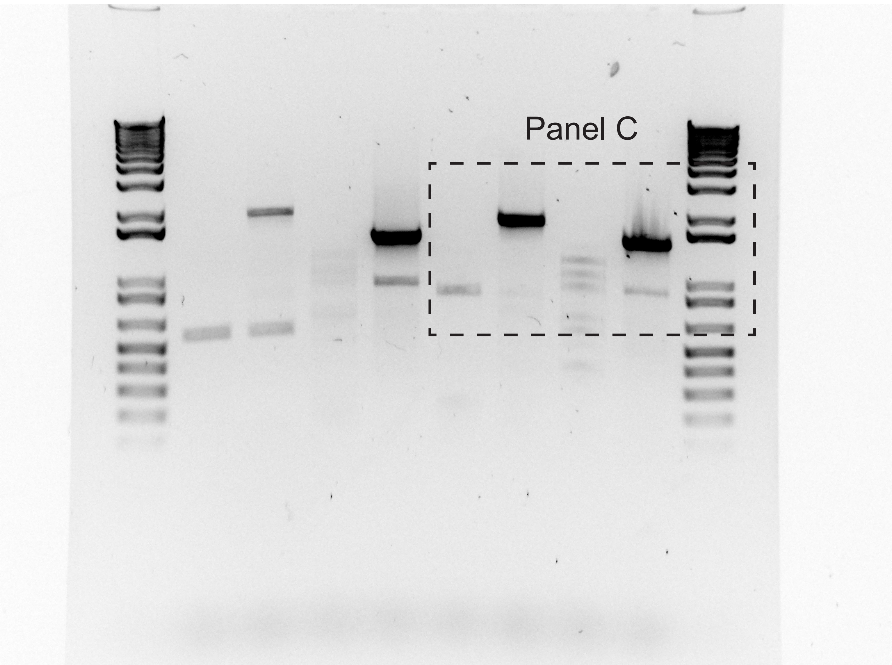

Inverted and increased levels

Figure 2-figure supplement 1-source data

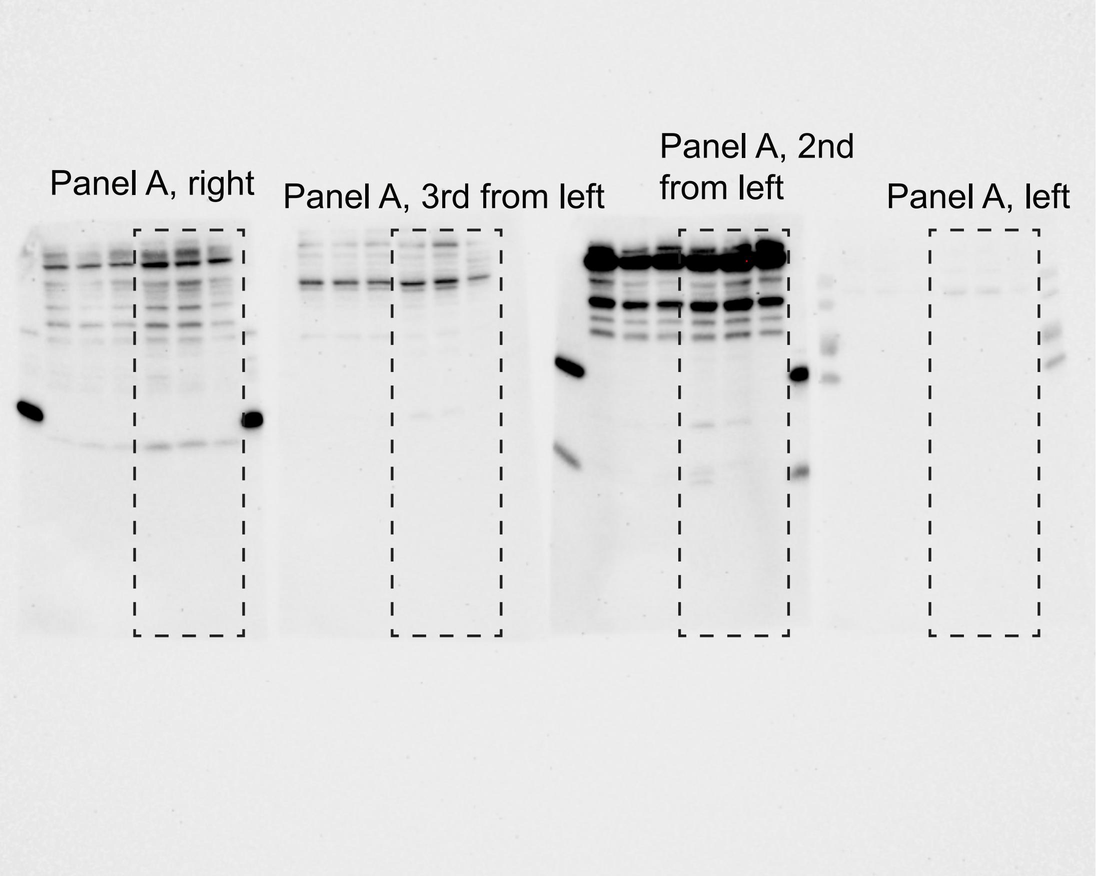

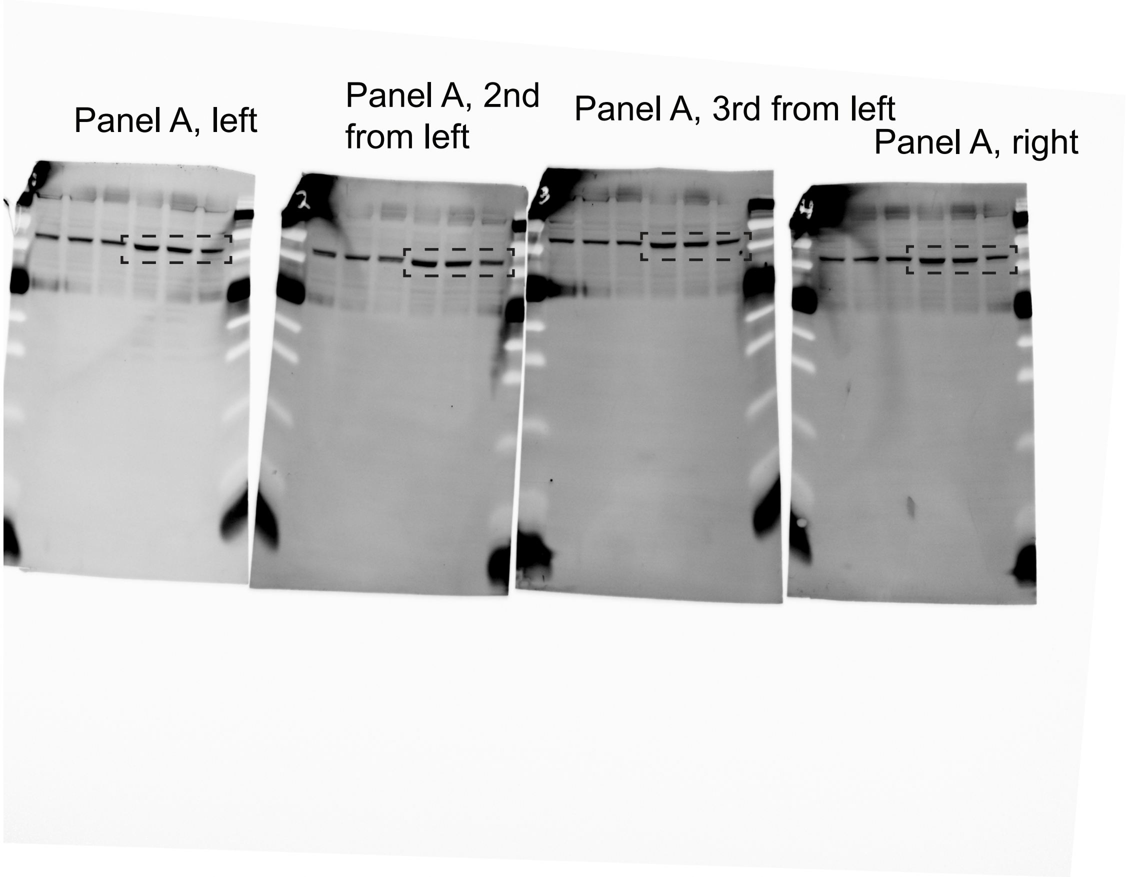

Figure 6-figure supplement 2-source data1-2

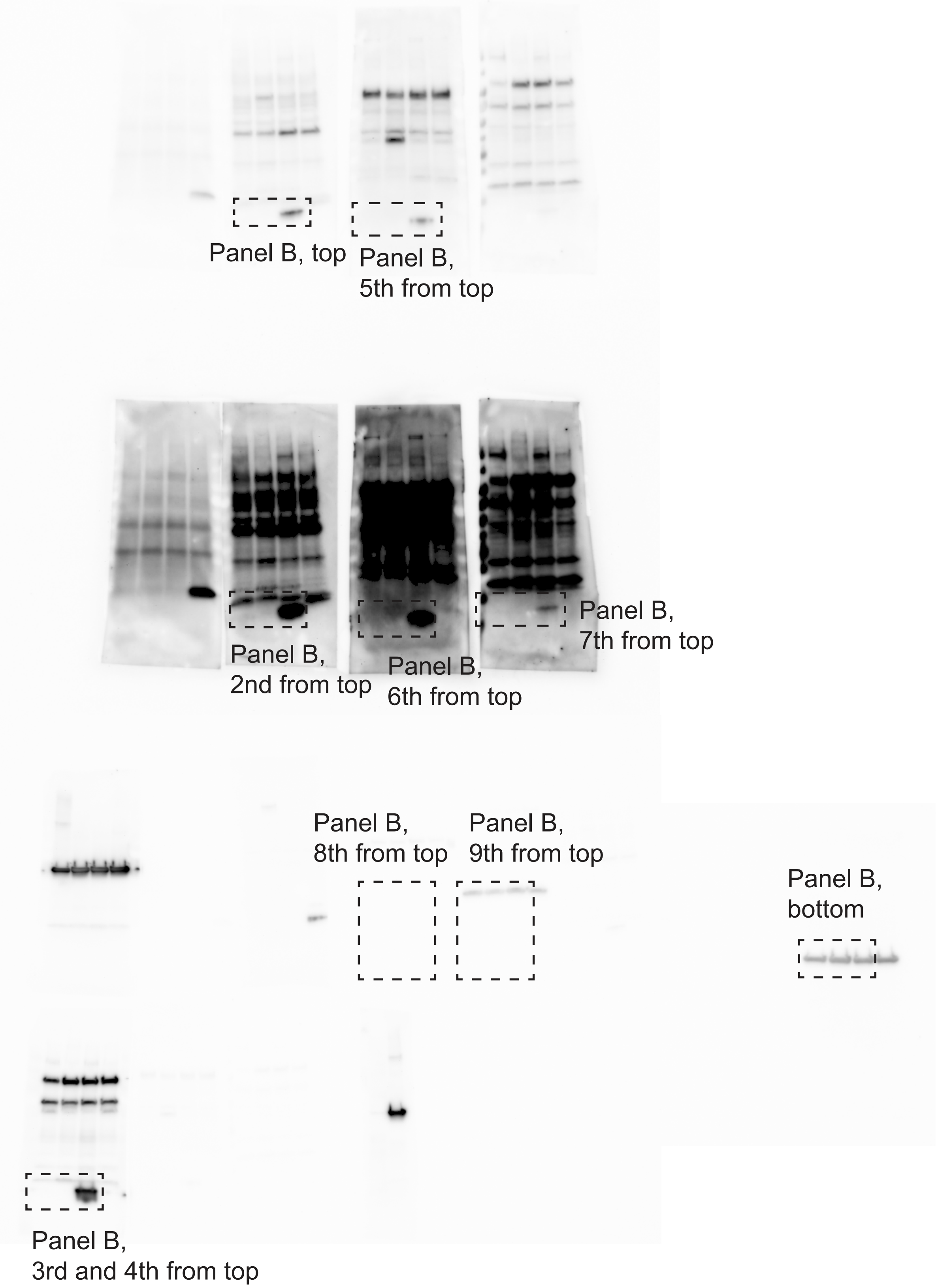

Figure 6-figure supplement 2-source data 3-6

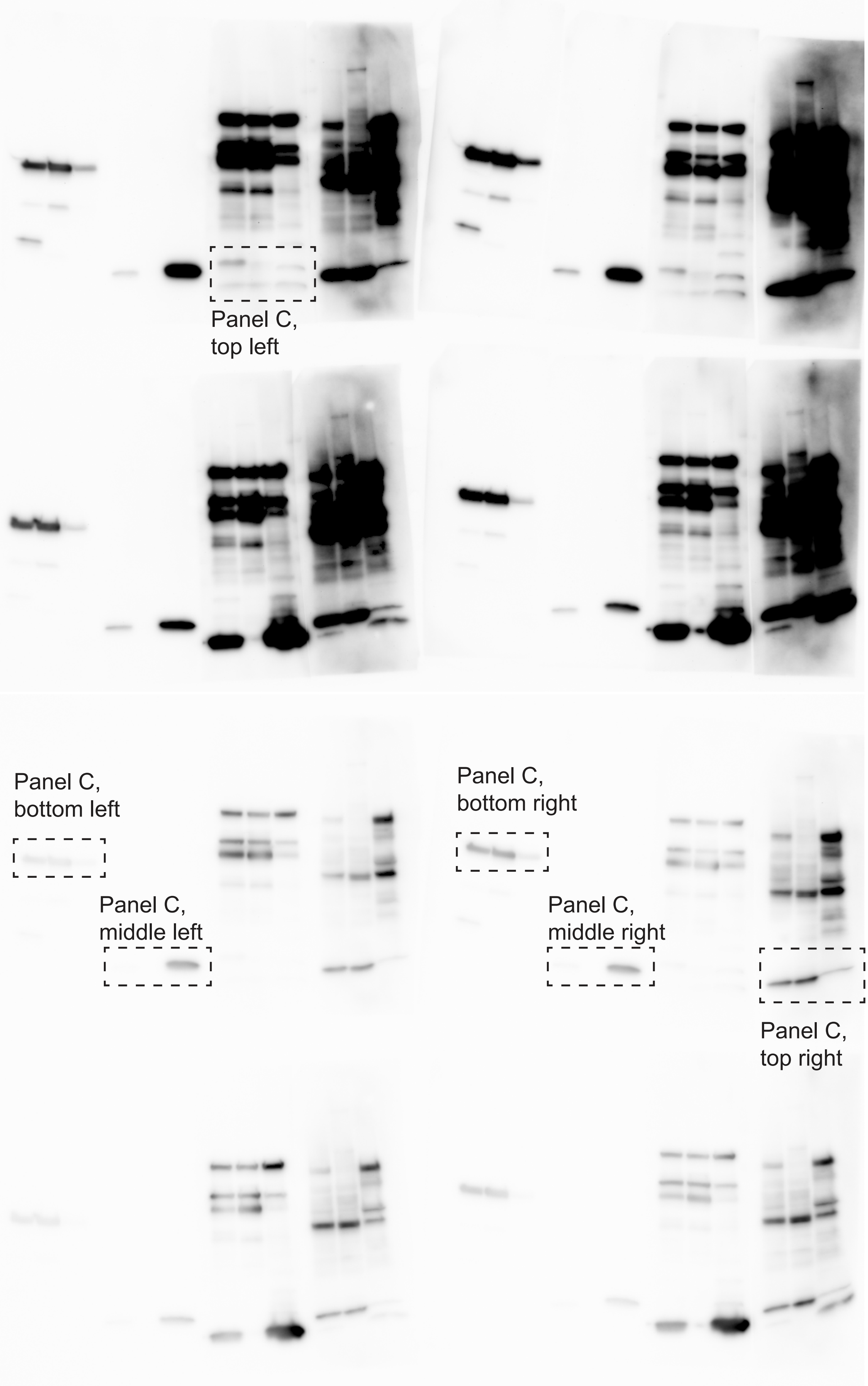

Figure 6-figure supplement 2-source data 7-8

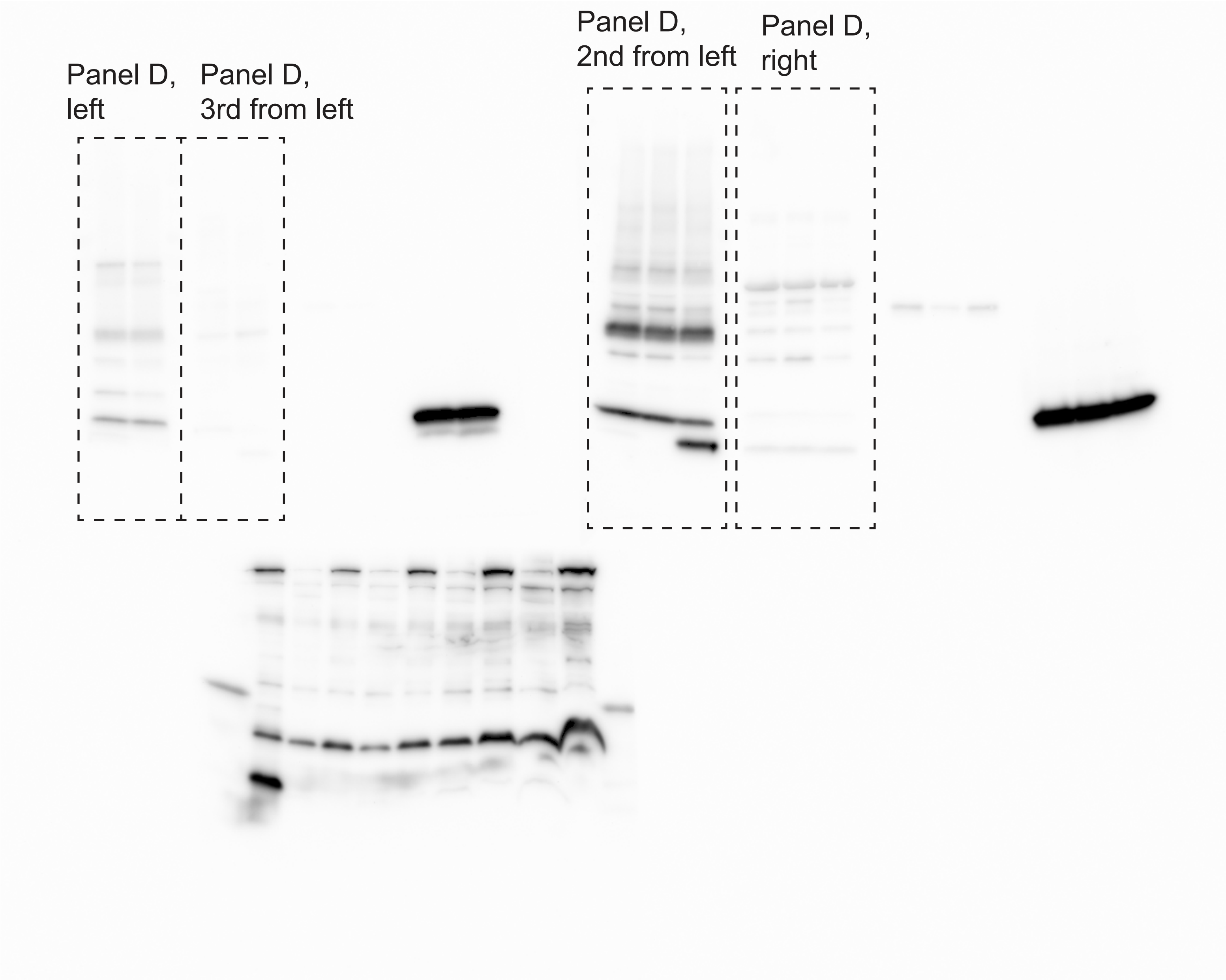

Figure 6-figure supplement 2-source data 9

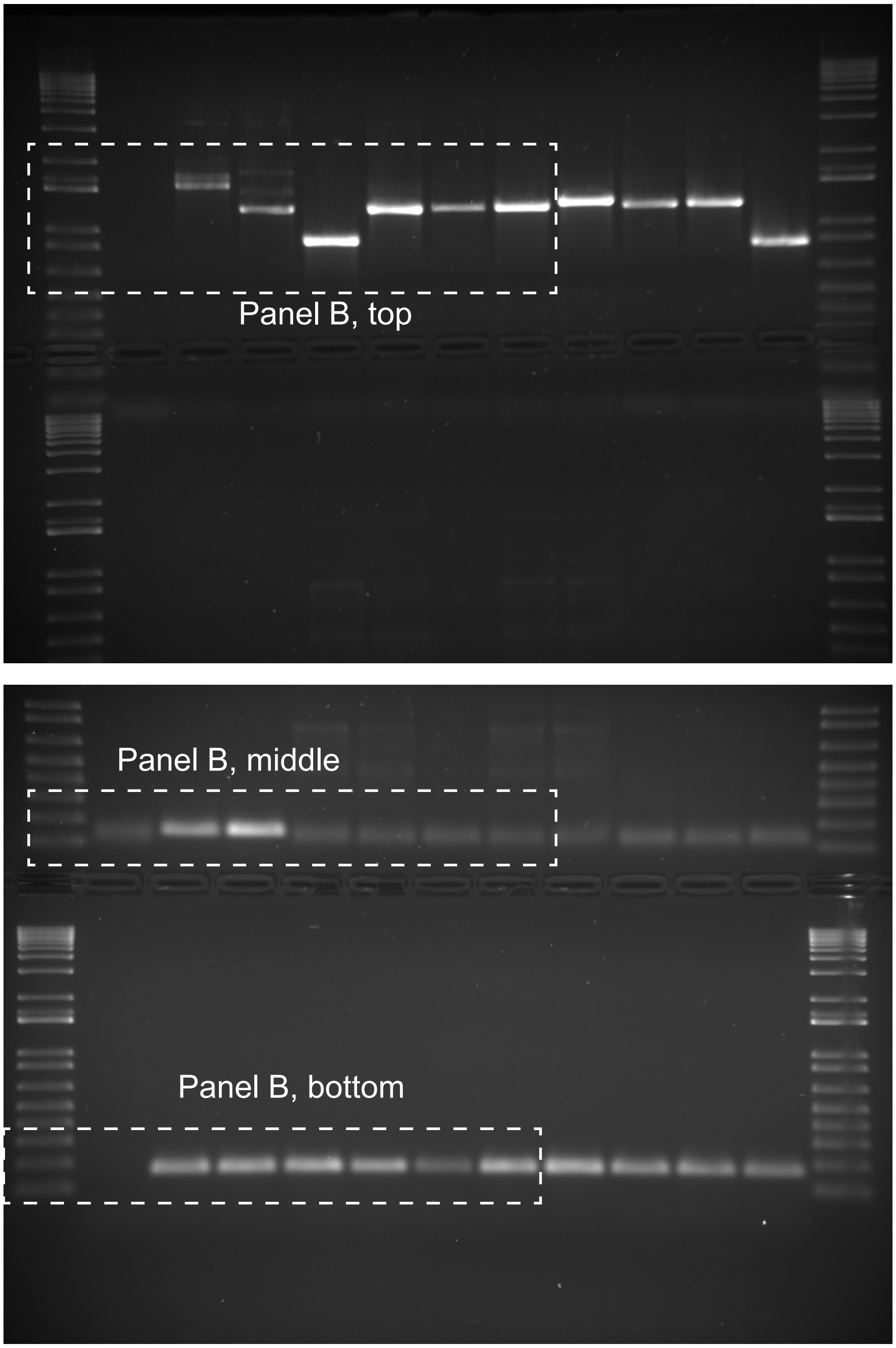

Figure 7-figure supplement 1-source data
